# Alteration in Inflammatory Responses and Cytochrome P450 Expression of Porcine Jejunal Cells by Drinking Water Supplements

**DOI:** 10.1155/2019/5420381

**Published:** 2019-01-02

**Authors:** Orsolya Palócz, Géza Szita, György Csikó

**Affiliations:** ^1^Department of Pharmacology and Toxicology, University of Veterinary Medicine, István u. 2., Budapest 1078, Hungary; ^2^Department of Food Hygiene, University of Veterinary Medicine, István u. 2, Budapest 1078, Hungary

## Abstract

The intestinal epithelium is the first determining barrier to the drugs administered *per os*. Cytochrome P450 (CYP) enzymes are substantial in the initial step of xenobiotic metabolism; therefore, intestinal CYP enzyme activities could be an important influencing factor of the oral utilization of xenobiotic substances. In this study, the effect of four drinking water supplements on CYP mRNA levels of porcine intestinal epithelial cells was examined. Further goal of the study is to describe the effect of these feed additives on the proinflammatory response of the LPS-treated enterocytes. The nontransformed porcine intestinal epithelial cells (IPEC-J2) were grown on six-well polyester membrane inserts. Cell cultures were treated with LPS (10 *μ*g/ml), *β*-glucan (5 and 50 *μ*g/ml), sanguinarine-containing additive (5 and 50 *μ*g/ml), drinking water acidifier (0.1 and 1 *μ*l/ml), and fulvic acid (25 and 250 *μ*g/ml) for 1 hour. Cells were washed with culture medium and incubated for additional 1 h before total RNA isolation. IL-6, IL-8, TNF-*α*, HSP70, CYP1A1, CYP1A2, and CYP3A29 mRNA levels were measured. The LPS treatment upregulated the gene expression of IL-8 and TNF-*α*. The relative gene expression of IL-6 remained unchanged and TNF-*α* and HSP70 were downregulated after the treatment with each feed additive. CYP1A1 and CYP1A2 expressions increased after sanguinarine-containing solution, fulvic acid, and drinking water acidifier treatment. None of the treatments changed the gene expression of CYP3A29, responsible for the metabolism of the majority of drug substances used in swine industry. The feed additive substances inhibited the expression of proinflammatory mediators HSP70 and TNF-*α*; however, *β*-glucan and fulvic acid elevated the production of the chemokine IL-8 mRNA in endotoxin-treated enterocytes. All acidic supplements increased the expression of CYP1A1 gene; their constituents may serve as a ligand of CYP1A1 nuclear receptors.

## 1. Introduction

The intestinal epithelium allows the absorption of water and nutrients and serves as a first barrier to microbes and also inducing and modulating immune responses [1]. The large monolayer surface comprised of the intestinal epithelial cells is also the first point of entry to the different orally applied drugs and other chemicals. The intestinal cytochrome enzymes play a key role in the initial step of xenobiotic metabolism.

Swine is one of the most important protein sources for humans; therefore, the food safety of feed and drinking water additives should be evaluated in this species. The noncancerous porcine intestinal epithelial cell line (IPEC-J2) originated from the jejunum is a good *in vitro* alternative and suitable model for preliminary investigations [2].

Various drinking water supplements are available on the market to improve production. We investigated the effect of fulvic acid, a sanguinarine-containing product, a drinking water acidifier, and *β*-glucan among them.

Fulvic acid is one of the most active fractions of humic substances which are commonly found in soil. Humic substances have been widely used in animal nutrition to improve the profitability of animal production and the health status of animals [3]. Fulvic acid molecule contains reactive functional groups, e.g., carboxyls, hydroxyls, carbonyls, phenols, and quinones, which are responsible for its metal chelating and antioxidant activity [4]. Previous studies indicated that fulvic acid formed a film on the mucus epithelium of the gastrointestinal tract, protected against infections and toxins, and improved utilization of nutrients in animal feed [5]. Dietary supplementation with fulvic acid improved feed efficiency and immunity as well [6].

The applied drinking water acidifier contains volatile fatty acids, amino acids, phosphoric acid, and zinc and copper salt complexes. The aqueous solution of the product contains undissociated organic acids, which inhibit the growth of pathogen microorganisms [7].

*β*-Glucans derived from fungi and yeast, which consist of a (1,3)-*β*-linked backbone with small numbers of (1,6)-*β*-linked side chains, are essentially known for their immune-modulating effects; oral delivery of them impacts mucosal immunity, as shown by an increase of intraepithelial lymphocytes in the intestine [8]. *β*-Glucan–mediated immunomodulatory effects on dendritic cell activation, macrophage phagocytosis, T helper cell, and cytotoxic T-lymphocyte priming and differentiation, and *in vivo* antitumor immune responses, are dependent of the dectin-1 pathway [9].

Sangrovit® is a natural plant-derived (*Macleaya cordata*) phytogenic feed additive containing many benzophenanthridine alkaloid compounds, the most abundant of which is sanguinarine [10]. Sanguinarine is used in swine, bovine, and poultry diets [11]. Quaternary benzophenanthridine alkaloids have been shown to have antimicrobial anti-inflammatory and immunomodulatory effects [12] and have selective effects on microbial growth along the digestive tract [13].

Bacterial products or metabolites alter the secretion pattern of mediators such as heat shock proteins (e.g., HSP70 and HSP90), proinflammatory cytokines (e.g., TNF-*α* and IL-6), and chemokines (e.g., IL-8/CXCL8) of intestinal epithelial cells [14, 15]. Permanent elevated level of inflammatory mediators can lead to impairment of intestinal signalling pathways, facilitate pathogen invasion, and emergence of disease [16].

The protective effect of four commercially available feed additives on endotoxin-induced inflammatory responses was examined; gene expression levels of characteristic effector molecules, IL-6, IL-8, TNF-*α*, and HSP70, were monitored in porcine intestinal cells. Furthermore, the impact of these test substances on the expression of cytochrome P450 genes was investigated to evaluate the possible first-pass effect of intestinal epithelium due to feed supplementation.

## 2. Materials and Methods

### 2.1. Applied Supplements

Bakers' yeast *β*-glucan (Wellmune WGP®, Lot: 14052-016, Biothera Company, USA; main component: water soluble *β*-(1-3), (1-6)-D-glucan; recommended dose: 5–10 mg/kg body weight).

Sanguinarine-containing product (Sangrovit® WS, Lot: 412a081, Phytobiotics Gmbh., Germany; main components: quaternary benzophenanthridine alkaloids and protopine alkaloids; recommended dose: 10–100 g/1000 l drinking water).

Drinking water acidifier (Immunofort®, Lot: 120915-035, Europharmavet Ltd., Hungary; main components: formic acid, propionic acid, phosphoric acid, methionine hydroxy analogue, zinc, and copper; recommended dose: 1 l/1000 l drinking water).

Fulvic acid (Fulvix pulvis®, Lot: 210515-012, Alpha-Vet Ltd./Organit Ltd., Hungary; main component: fulvic acid: 70%; recommended dose: 250 g/1000 l drinking water).

### 2.2. Cell Line and Culture Conditions

The nontransformed porcine intestinal epithelial cell line IPEC-J2, originally isolated from jejunal epithelia of a neonatal unsuckled piglet [17], was a kind gift of Dr. Jody Gookin, Department of Clinical Sciences, College of Veterinary Medicine, North Carolina State University, Raleigh, NC, USA. IPEC-J2 cells were maintained on six-well Transwell polyester membrane inserts (Corning Inc., Corning, NY, USA) as it was described previously [18]. Transepithelial electrical resistance (TEER) measurement of monolayers was performed on alternate days after seeding, from day 5 to 21 of culture, using an EVOM Epithelial Tissue Volt/Ohmmeter (World Precision Instruments, Berlin, Germany).

### 2.3. Neutral Red Uptake Assay for Cell Viability

Influence of lipopolysaccharide (LPS), sanguinarine-containing product (SN), drinking water acidifier (DWA), *β*-glucan, and fulvic acid on the viability of enterocytes was tested. The substances were dissolved and diluted in cell culture medium. IPEC-J2 cells were seeded onto a 96-well plate and incubated with the test substances for 1, 4, and 24 h. Viability of IPEC-J2 cells was measured 24 hours after treatment by neutral red uptake assay as described by Repetto et al. [19].

### 2.4. Treatment of Cell Cultures

Before treatment, confluent monolayers of the IPEC-J2 cells were washed with plain medium (Dulbecco's Modified Eagle Medium : F12; Lonza Verviers Sprl., Belgium). Untreated control IPEC-J2 cell cultures received plain medium without supplementation of the test materials. LPS and the other solutions were prepared freshly prior to each experiment. LPS was added in plain medium at 10 *μ*g/ml on the apical side of the IPEC-J2 layer. The other substances were added in plain medium alone or simultaneously with LPS in the following concentrations: sanguinarine-containing product (SN) (5 and 50 *μ*g/ml), drinking water acidifier (DWA) (0.1 and 1 *μ*l/ml), *β*-glucan (5 and 50 *μ*g/ml), and fulvic acid (25 and 250 *μ*g/ml). The above concentrations were selected corresponding to the manufacturer's recommendation; the higher doses of each supplement concur to the recommended dose, and the 10-fold dilutions represent the lower doses. Plain medium was added to the basolateral side of the cells and both to the apical and basolateral compartment of the control wells. After 1 h incubation with LPS and the other substances, cells were washed with plain medium and cultured for additional 1 h for PCR studies. TEER measurements were performed both before and after the LPS treatment.

### 2.5. Quantitative Real-Time PCR

One hour after the treatment, culture medium was removed and 1 ml of ice-cold RNAzol RT reagent (Sigma-Aldrich, Steinheim, Germany) was added to the IPEC-J2 samples. The additional one hour incubation time was kept, before the cells were collected, for gaining the highest gene expression level according to previous studies [20]. Samples were collected and kept at −80°C until further processing. Total RNA was isolated from the cells according to the manufacturer's instructions. Quantity, A260/A280 and A260/A230 ratios of the extracted RNA were determined using a NanoDrop ND-1000 Spectrophotometer (Thermo Scientific, Wilmington, USA).

Synthesis of the first strand of cDNA from 1000 ng of total RNA and quantitative real-time PCR (qPCR) was performed according to Palócz et al. [18]. Tested genes of interest were IL-6, IL-8, TNF-*α*, CYP1A1, CYP1A2, CYP3A29, and HSP70. Hypoxanthine phosphoribosyl transferase (HPRT) and cyclophilin-A (CycA) were used as reference genes. Primer sequences are listed in Table S1 in Supplementary Materials.

### 2.6. Statistical Analyses

Relative gene expression levels of the gene of interests were calculated by the relative expression software tool (REST) 2009 software. Statistical analyses were performed by Statistica 13 software (Dell Inc., Round Rock, USA). Differences between means were evaluated by one-way analysis of variance (ANOVA) followed by a post hoc comparison using Fisher's least significant difference (LSD) test.

## 3. Results

### 3.1. Cell Viability Test

Viability of the cells was monitored after LPS (10 *μ*g/ml), SN (5 and 50 *μ*g/ml), DWA (0.1 and 1 *μ*l/ml), *β*-glucan (5 and 50 *μ*g/ml), and fulvic acid (25 and 250 *μ*g/ml) treatment. Viability of the IPEC-J2 cells was not altered after 1, 4, and 24 h of the treatment with each test substance.

According to the measured TEER of the cell cultures before and after the treatments (see Table S2 in Supplementary Materials), the integrity of the monolayer IPEC-J2 cell cultures was not damaged due to the 1-hour treatment with the test substances.

### 3.2. Effect of Feed Additives on the Relative Expression of Inflammatory Genes after LPS Treatment

**Figure 1:**
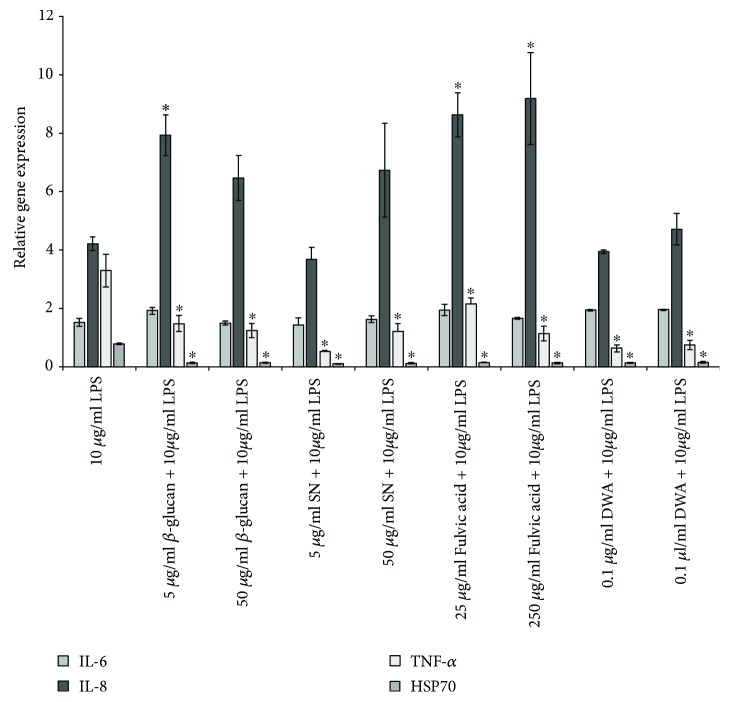
Relative gene expression (mRNA) of IL-6, IL-8, TNF-*α*, and HSP70 of IPEC-J2 cells exposed to LPS treatment (at 10 *μ*g/ml, 1 h) compared to untreated controls (*n* = 6/group). Significant differences are shown in comparison to LPS treatment (^∗^ *p* < 0.05) Data are shown as means±SEM. LPS: lipopolysaccharide; SN: sanguinarine-containing product; DWA: drinking water acidifier; IL: interleukin; TNF-*α*: tumour necrosis factor alpha; HSP70: heat shock protein 70.

The one-hour treatment with 10 *μ*g/ml LPS upregulated the gene expression of IL-8 and TNF-*α* compared to untreated controls. The relative gene expression of IL-6 remained unchanged, and TNF-*α* and HSP70 were downregulated after the treatment with each supplement compared to the LPS-treated group. The simultaneous LPS and either *β*-glucan or fulvic acid treatments resulted in increased IL-8 mRNA level compared to LPS-treated cells (Figure 1).

### 3.3. Effect of Feed Additives on the Relative Expression of CYP450 Genes

**Figure 2:**
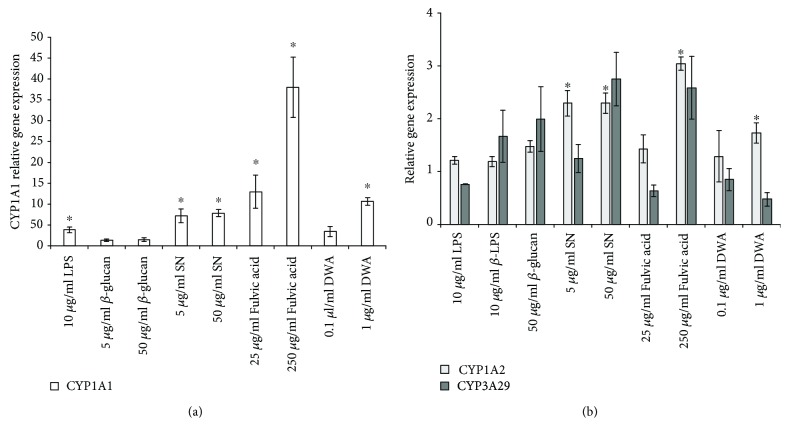
Relative expression of CYP1A1 (a) and CYP1A2 and CYP3A29 (b) genes in feed additive treated IPEC-J2 cells compared to untreated groups (*n* = 6/group, ^∗^ *p* < 0.05). Data are shown as means ± SEM. LPS: lipopolysaccharide; SN: sanguinarine-containing product; DWA: drinking water acidifier; CYP: cytochrome P450.

CYP1A1 expression increased after one-hour LPS, SN, fulvic acid, and high-dose DWA treatment. CYP1A2 mRNA level enhanced following the treatment with both doses of SN, high-dose fulvic acid, and high-dose DWA (Figure 2). None of the treatments changed CYP3A29 gene expression. The *β*-glucan treatment did not alter any of the CYP450 mRNA levels.

## 4. Discussion

The drinking water supplements prevented the upregulation of the proinflammatory cytokine TNF-*α* in LPS-treated enterocytes. Together with LPS, the *β*-glucan and fulvic acid treatment further enhanced the level of IL-8 mRNA. This equivocal act may be explained by the different functions of the mediators. Unlike the proinflammatory cytokine TNF-*α* that activates inflammatory mediators, IL-8 is classified as chemokine, and its main effector role is to recruit neutrophils to the site of inflammation [21, 22]. Increased IL-8 and decreased TNF-*α* may prevent the body from systemic damage due to chronic inflammation. Elevated IL-8 (CXCL8) level results in only transient pyrexia and less pathogen bacteria shedding of *Salmonella*-infected pigs while increased TNF-*α* level can lead to prolonged inflammatory condition and persistent pathogen shedding [23]. Lower pathogen burden is a consequence of a more specific inflammatory response resulting in a better general condition of the animals. HSP70 was downregulated after the simultaneous treatment with LPS and the supplements. Heat shock proteins have been reported to stimulate the production of proinflammatory cytokines such as TNF-*α*, IL-1, IL-6, and IL-12 [24]; decreased HSP70 level may lead to reduced proinflammatory cytokine level. Due to suppressed inflammation, the animals could utilize the saved energy to gain weight and achieve better performance. The immunomodulatory effect of *β*-glucan, SN, fulvic acid, and DWA in enterocytes was demonstrated *in vitro*, although these results should be substantiated by live animal studies.

The majority of the veterinary drugs are decomposed through the CYP3A29 enzyme in swine [25], which was not influenced by any of the treatments; consequently, their concomitant use with veterinary medicinal substances is safe. However, the enhanced CYP mRNA levels at higher doses of the supplements call the attention of the importance of the accuracy of dosing. The relative gene expression of CYP1A1 was substantially elevated (35 times) due to the high-dose fulvic acid treatment, which presumes that the fulvic acid molecule may be a high affinity ligand of a nuclear receptor of the CYP1A1. It was demonstrated in rat hepatoma cells that humic substances can induce the aryl-hydrocarbon receptor [26]. Interestingly, all test substances which have acidic characteristic enhanced the gene expression level of CYP1A1; it is possible that all of them contain such constituents, which are ligands of nuclear receptor of CYP1A1 gene, e.g., short-chain fatty acids in DWA and protopine alkaloids and quaternary benzophenanthridine alkaloids in SN. Nonetheless, according to Vrba et al. [27], protopine alkaloids increased both CYP1A1 and CYP1A2 gene expression in human hepatocytes through AhR independent mechanism. The increased level of CYP1A1 and CYP1A2 enzymes may cause elevated biotransformation of drugs used in swine industry, e.g., azole antifungals, flubendazole, fenbendazole [28], quinolone antimicrobials, danofloxacin, marbofloxacin, and enrofloxacin [29]. Among the tested substances, *β*-glucan appears to be the safest additive in terms of drug metabolism since it is not exerted any effect on the investigated CYP genes.

## 5. Conclusions

In conclusion, the administration of the investigated additives in parallel with veterinary pharmaceuticals is safe because the level of CYP3A29 mRNA was not affected by any of the treatments. Although the supplements with acidic properties increased the intestinal gene expression of certain CYP genes, especially at higher doses, consequently, it may lead to elevated CYP protein level, potentiating possible feed-drug interactions, in particular with those substances which are metabolized via the CYP1A subfamily. Although their stimulating effect on the CYP enzymes should be supported by *in vivo* studies, CYP protein levels should be monitored. The inhibitory effect of all tested feed additives on proinflammatory cytokines was demonstrated in endotoxin-evoked inflammation, although the chemokine IL-8 production was enhanced or remained unchanged after simultaneous LPS and feed supplement treatments. Increased IL-8 and suppressed TNF-*α* may lead to a more specific inflammatory response in the presence of pathogens and consequently prevent chronic inflammation.

## Data Availability

The data used to support the findings of this study are available from the corresponding author upon request.

## Supplementary Materials

Supplementary Materials Table S1: sequence of primer sets used for quantitative real-time PCR analysis. Table S2: transepithelial electrical resistance (TEER) values between apical and basolateral compartment of IPEC-J2 cell cultures. Average and standard deviation (SD) of TEER (Ohm × cm^2^) data are shown before the treatments and after one-hour incubation time with the test substances [30–37].
