# Supplementary material for: Alteration in Inflammatory Responses and Cytochrome P450 Expression of Porcine Jejunal Cells by Drinking Water Supplements
Source: Mediators Inflamm. 2019 Jan 2;2019:5420381. doi: 10.1155/2019/5420381 (PMC6334367; doi:10.1155/2019/5420381)
Supplement: Supplementary Materials — Table S1: sequence of primer sets used for quantitative real-time PCR analysis. Table S2: transepithelial electrical resistance (TEER) values between apical and basolateral compartment of IPEC-J2 cell cultures. Average and standard deviation (SD) of TEER (Ohm × cm2) data are shown before the treatments and after one-hour incubation time with the test substances [30–37]. [file 5420381.f1.pdf]

Table S1. Sequence of primer sets used for quantitative real-time PCR analysis

Gene symbol	Accession number	Primer sequences (5'-3')	Product size (bp)	Efficiency	Reference
IL-8	NM_213867	F: AGAGGTCTGCCTGGACCCCA<br>R: GGGAGCCACGGAGAATGGGT	126	1.972	[30]
IL-6	NM_214399	F: TTCACCTCTCCGGACAAAAC<br>R: TCTGCCAGTACCTCCTTGCT	122	1.970	[31]
TNF-α	NM_214022	F: TTCCAGCTGGCCCCTTGAGC<br>R: GAGGGCATTGGCATACCCAC	146	1.873	[32]
CYP1A1	NM_214412.1	F: CAGAGCTGCTTAGCCTTATCAACC<br>R: CTGGATGCTGGGATTTGTCACCAG	386	2.00	[33]
CYP1A2	NM_001159614.1	F: GTGAGGAGATGTTCAGCATCGTGAAG<br>R: CTTCTGTATCTCAGGATATGTCACA	386	1.750	[34]
CYP3A29	NM_214423.1	F: TTCGTGCTTCACAGAGAGACCC<br>R: TACTAGGTGGGGGTGGATGG	576	1.975	[35]
HSP70	NM_001123127	F: GCCCTGAATCCGCAGAATA<br>R: TCCCCACGGTAGGAAACG	152	2.0	[36]
CycA	NM_214353	F: GCGTCTCCTTCGAGCTGTT<br>R: CCATTATGGCGTGTGAAGTC	160	1.907	[32]
HPRT	NM_001032376	F: GGACTTGAATCATGTTTGTG<br>R: CAGATGTTTCCAAACTCAAC	91	1.963	[37]

F: Forward, R: Reverse, IL: Interleukin, TNF-α: Tumour necrosis factor alpha, CYP: Cytochrome P450, HSP70: Heat shock protein 70, HPRT: Hypoxanthine phosphoribosyl transferase, CycA: Cyclophilin-A

Table S2. Transepithelial electrical resistance (TEER) values between apical and basolateral compartment of IPEC-J2 cell cultures. Average and standard deviation (SD) of TEER (Ohm×cm^2) data are shown before the treatments and after one-hour incubation time with the test substances.

	Average of TEER (Ohm×cm^2) prior to treatment	SD of TEER (Ohm×cm^2) prior to treatment	Average of TEER (Ohm×cm^2) after treatment	SD of TEER (Ohm×cm^2) prior to treatment
Control	6712	211	6664	1011
10 µg/ml LPS	6547	60	6586	386
5 µg/ml β-glucan	6910	62	6870	1128
50 µg/ml β-glucan	6910	216	7352	238
5 µg/ml SN	6280	59	7400	645
50 µg/ml SN	6367	209	6784	141
25 µg/ml Fulvic acid	6580	262	7355	321
250 µg/ml Fulvic acid	6800	481	7041	203
0.1 µl/ml DWA	7639	16	7382	598
1 µl/ml DWA	7769	255	7718	403
5 µg/ml β-glucan + 10 µg/ml LPS	7103	182	4592	441
50 µg/ml β-glucan + 10 µg/ml LPS	7302	185	5132	728
5 µg/ml SN + 10 µg/ml LPS	6849	183	5075	606
50 µg/ml SN + 10 µg/ml LPS	7031	76	5283	730
25 µg/ml Fulvic acid + 10 µg/ml LPS	6824	105	6127	1090
250 µg/ml Fulvic acid + 10 µg/ml LPS	6972	386	8418	12
0.1 µl/ml DWA + 10 µg/ml LPS	7709	300	5940	521
1 µl/ml DWA + 10 µg/ml LPS	7838	236	6083	874

LPS: Lipopolysaccharide, SN: Sanguinarine containing product, DWA: Drinking water acidifier

The integrity of the monolayer IPEC-J2 cell cultures was not damaged due to the 1 hour treatment with the test substances.
